# Spatial selective manipulation of microbubbles by tunable surface acoustic waves

**DOI:** 10.1063/1.4954934

**Published:** 2016-06-28

**Authors:** Wei Zhou, Lili Niu, Feiyan Cai, Fei Li, Chen Wang, Xiaowei Huang, Jingjing Wang, Junru Wu, Long Meng, Hairong Zheng

**Affiliations:** 1 Paul C. Lauterbur Research Center for Biomedical Imaging, Institute of Biomedical and Health Engineering, Shenzhen Institutes of Advanced Technology, Chinese Academy of Sciences, Shenzhen 518055, People's Republic of China; 2 Research Center for Micro/Nano Systems and Bionic Medicine, Institute of Biomedical and Health Engineering, Shenzhen Institutes of Advanced Technology, Chinese Academy of Sciences, Shenzhen 518055, People's Republic of China; 3 Department of Physics, University of Vermont, Burlington, Vermont 05405 USA

## Abstract

A microfluidic device based on a pair of slant-finger interdigital transducers (SFITs) is developed to achieve a selective and flexible manipulation of microbubbles (MBs) by surface acoustic waves (SAWs). The resonance frequency of SAWs generated by the SFITs depends on the location of its parallel pathway; the particles at different locations of the SAWs' pathway can be controlled selectively by choosing the frequency of the excitation signal applied on the SFITs. By adjusting the input signal continuously, MBs can be transported along the acoustic aperture precisely. The displacement of MBs has a linear relationship with the frequency shift. The resolution of transportation is 15.19 ± 2.65 *μ*m when the shift of input signal frequency is at a step of 10 kHz. In addition, the MBs can be controlled in a two-dimensional plane by combining variations of the frequency and the relative phase of the excitation signal applied on the SFITs simultaneously. This technology may open up the possibility of selectively and flexibly manipulating MBs using a simple one-dimensional device.

## I. INTRODUCTION

Manipulation of particles plays a critical role in the microfluidic technology and it has been used in various fields, such as gene expression,^1^ clinical medicine,^2^ drug development,^3^ environment,^4^ and food hygiene technologies.^5^ Several methods have been utilized to transport particles under an external force field, such as optical tweezers,^6^ magnetic tweezers,^7^ dielectrophoresis force,^8^ hydrodynamic,^9^ and acoustic methods.^10,11^ Due to the noninvasive and non-contact characteristics, acoustic manipulation technology has been shown to be advantageous in controlling the movement of particles at the microscopic scale.

Both bulk waves and surface acoustic waves (SAWs) are widely used in acoustic manipulation techniques. For the bulk wave, by switching on and off the active elements along the piezoelectric array, particles can be moved in one dimension.^12^ A multi-element bulk wave transducer was used to generate acoustic vortices^13^ which can move particles in a dexterous fashion. Recently, SAWs devices received increasing attention in microfluidic system because the device can be miniaturized, and acoustic wave can be localized along the solid–liquid interface.^14–16^ As a versatile method for particle manipulation, SAWs based microfluidic devices have been used to manipulate various objects, such as polystyrene micro-spheres,^17–19^ cells,^16,20–22^ and organisms.^23^ The traveling SAWs with the low power consumption is developed to separate various particles in size through a Schröder diffuser.^24^ Guo *et al.* have demonstrated that a controllable motive force generated by SAWs can be used to precisely control the intercellular distance.^25^ Furthermore, bioeffects on living tissues induced by SAWs can be readily investigated at cellular and molecular levels.^26–28^

The encapsulated gas-filled microbubbles (MBs), known as ultrasound contrast agents, range from 0.5 *μ*m to 10 *μ*m in diameter and are very sensitive to the ultrasound irradiation, which was originally designed to improve the contrast quality of ultrasound images.^29^ With the development of the MBs, the molecular probes, such as tissue-specific drugs and genes, can be coupled to the shell of MBs, enabling the realization of ultrasound molecular imaging and targeted drug delivery.^30,31^ Thus, manipulation of MBs has attracted increasing attention from a wide range of scientific interests.^32–35^

MBs in an acoustic field experience the acoustic radiation force and could be driven to move due to the acoustic impedance mismatch between the MBs and the fluid medium, regardless of MBs' optical, electronic, and magnetic properties. All the MBs in the acoustic field perform the essentially same movement, and therefore acoustic manipulation has emerged as a powerful tool for massively parallel manipulation of MBs and living cells. However, for the same reason, it is difficult to control specific MBs at a spatial location to realize the selective manipulation.

**FIG. 1.**
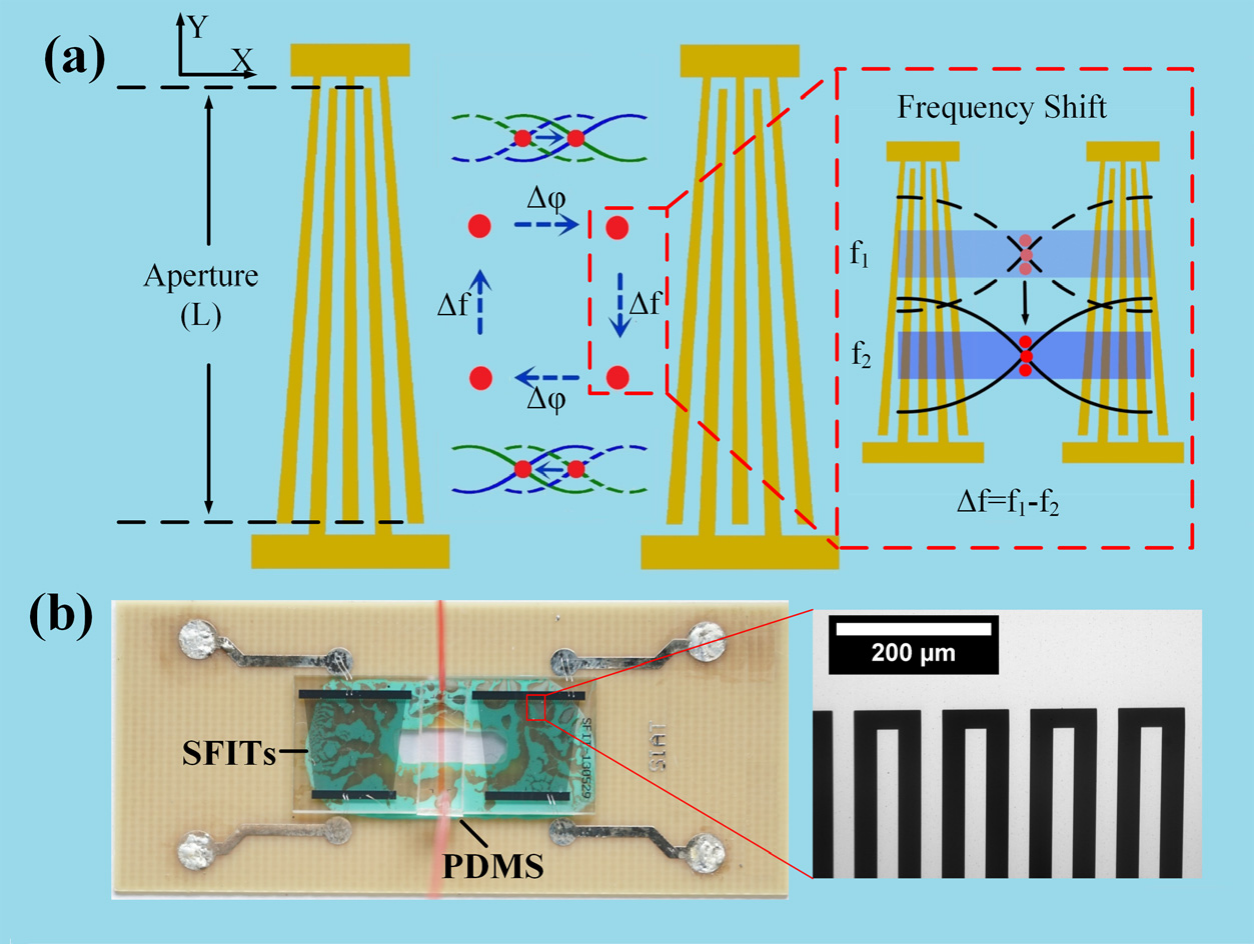
(a) A schematic illustration of selective and flexible manipulation of microbubbles (MBs) by SAWs. A microfluidic device consists of a pair of SFITs and a PDMS microchannel. MBs at a specific location can be handled by exciting the corresponding resonant frequency and can also be transported in X and Y directions by adjusting the relative phase and the resonant frequency simultaneously. (b) The device consisting of SFITs and a PDMS channel. Inset: the width of the interdigital fingers changes continuously.

In this letter, a microfluidic device with a pair of slant-finger interdigital transducers^36^ (SFITs) has been developed to achieve the selective and flexible manipulation by SAWs. A particle's movement at the specific location can be controlled selectively using its resonant frequency corresponding to its location and the MBs can be transported along the Y direction by adjusting the driving frequency of the SFITs as shown in Fig. 1(a). Fig. 1(b) is an actual picture showing the SFITs device attached with polydimethylsiloxane (PDMS) microchannel. Furthermore, flexible and dynamic manipulation in two dimensions can also be performed in this microfluidic device by a combination of the phase-shift and frequency-shift methods.

## II. METHODS AND MATERIAL

### A. Working mechanism

The principle of the manipulation of MBs is shown in Fig. 1(a). A pair of SFITs is fabricated in parallel to each other and generates a one-dimensional standing surface acoustic wave (SSAW) along X direction. The acoustic radiation force acting on MBs in a standing acoustic field is equal to the negative gradient of acoustic pressure times MBs volume.^37^ Acoustic radiation force acting on MBs can be expressed as^38^

$$F_{b}=–〈V(t)\nabla P(r,t)〉=\frac{\pi \rho {\left|A\right|}^{2}kR}{1-\omega_{0}^{2}/\omega^{2}}\text{sin}(2kd),$$ (1)

where V(t) is the MBs volume at time t and P(r,t) is the acoustic pressure at position r at time t. ρ,A,k,andR are fluid density, wave amplitude, wave number, and bubble radius, respectively. d is the distance from MBs to nearest pressure nodes. $\omega \;\text{and}\;\omega_{0}$ are acoustic frequency and resonant frequency of MBs, respectively. In a standing wave field, the pressure nodes and pressure antinodes represent the position of the pressure minimum and maximum, respectively. The equilibrium location of MBs (nodes or antinodes) in a standing acoustic field depends on the resonance frequency of MBs and the acoustic frequency. Since the resonant frequency of MBs is much lower than the lowest resonant frequency of SFITs (17.10 MHz), most of the MBs will be trapped at pressure nodes.^39^

Because of the continuous variation of the width of finger electrodes, the resonance frequency of SFITs changes linearly along the acoustic aperture. When adjusting the input signal frequency, the acoustic path of SAWs generated by SFITs will be changed along the acoustic aperture, correspondingly, as shown in Fig. 1(a). As there is a linear relationship between the shift of the resonant frequency and the position of the resonant location of SFITs, the transportation distance induced by frequency-shift can be expressed as^40,41^

$$\Delta y=\frac{\Delta y}{f_{\max}-f_{\min}}L,$$ (2)

where Δf represents the shift of input frequency, $f_{\max}\;\mathrm{and}\;f_{\min}$ represent the maximum and minimum resonance frequency of transducers, respectively, and L is the length of the acoustic aperture.

By adjusting the relative phase between the two transducers, the pressure nodes can be shifted correspondingly in the direction of acoustic propagation. A linear relationship between relative phase-shift and linear translation of pressure nodes can be expressed as^33^

$$\Delta x=\left(n-1\right)\frac{\lambda }{2}+\frac{\lambda }{720\circ }\varphi_{x}\;\;\;\;\varphi_{x}\in \left[0\circ ,360\circ \right]\;\;\;\;\;\;n=1,2,3.....,$$ (3)

where Δx represents the displacement of pressure nodes in X direction. n is the number of repetition of relative phase-shift from 0° to 360°, λ is the wavelength of SAWs, and $\varphi_{x}$ is the relative phase-shift between two transducers. It can be seen that the displacement of the particles has a good linear relationship with the shift of the driving frequency and the relative phase in the Y and X directions, respectively. By simultaneously adjusting the relative phase and the driving frequency between one-dimensional SFITs continuously, the particles can be transported precisely and moved along any trajectory in a plane. Furthermore, due to the relationship between the resonant frequency and the resonant location of SFITs, it is possible to manipulate a specific particle in the channel selectively by applying the corresponding driving frequency to SFITs.

### B. SAWs-PDMS microfluidic device

As show in Fig. 1(b), the microfluidic device consists of a pair of SFITs. The aluminum SFITs film with the thickness of 500 nm was sputtered on a 128° Y-cut lithium niobate substrate. To test the performance of SFITs, the device was glued to a printed circuit board (PCB) by sound absorber material, and the radio frequency (RF) signal could be applied to SFITs through the bonding wire connected to PCB. The period of SFITs varied gradually from 177 *μ*m to 229 *μ*m and their corresponding resonant frequencies changed from 22.00 MHz to 17.10 MHz along the acoustic aperture with a width of 8 mm.

The polydimethylsiloxane (PDMS, Sylgard184, Dow Corning, USA) channel was fabricated by a standard soft-lithograph and mold-replica process.^42^ PDMS mixed at a 10:1 curing ratio was poured into molds and subjected to a post bake at 80 °C for 40 min. Subsequently, the PDMS channel was peeled from the molds. The width and the height of the microchannel were 500 *μ*m and 50 *μ*m, respectively. A punch with a diameter of 0.75 mm (Harris Uni-Core, Ted Pella Inc., Redding, CA, USA) was used to drill holes for inlets and outlets. Both PDMS channel and lithium niobate substrate were subjected to plasma treatment (150 W, 3 min) and then the PDMS channel was adhered on the substrate to obtain a permanent bonding. The integrated device was then cured at 80 °C for 12 h to improve the bonding strength.

### C. Preparation of materials

MBs were chosen to demonstrate the performance of the microfluidic device since the response of MBs to acoustic wave is sensitive and the behavior of MBs in acoustic field is similar to the cells.^43^ The octafluoropropane (C_3_F_8_) gas-filled MBs were fabricated in-house by the mechanical agitation method.^44^ The diameters of MBs ranged from 0.5 *μ*m to 10 *μ*m, measured by a particle size analyzer (AccuSizer 780A; Particle Sizing Systems, Santa Barbara, CA, USA). The mean diameter of MBs was approximately 1.38 *μ*m. The monodisperse spherical beads (Sigma-Aldrich, St. Louis, MO, USA) were selected to prove the ability of selective manipulation as they have the same material (polystyrene) and diameter (10 *μ*m). Polystyrene beads were diluted with deionized water at a ratio of 1:100, and the final concentration was 4 × 10^6^ particles/ml measured by an automatic particle size analyzer.

### D. Experimental system

A microscope (D-35578 Wetzlar, Leica, Germany) was coupled with a high speed charge-coupled-device (CCD) camera (OptiMOS, QImaging, Canada) to record the behavior of particles. MBs and polystyrene beads were injected into a microchannel by a micro-syringe pump (Pump 33; Harvard Apparatus, South Natick, MA). A dual-channel arbitrary signal generator (AFG3102, Tektronix, USA) was used to generate sinusoidal signals. The RF signals were then amplified by power amplifier (Minicircuits ZHL-1–2W, Brooklyn, NY, USA) and eventually connected to SFITs. The input power applied to the single SFITs ranged from 20 mW to 220 mW, measured by a power sensor (Minicircuits PWR-4GHS, Brooklyn, NY, USA) with a cold 30 dB attenuator.

## III. RESULTS AND DISCUSSION

### A. Characteristics of SFITs

**FIG. 2.**
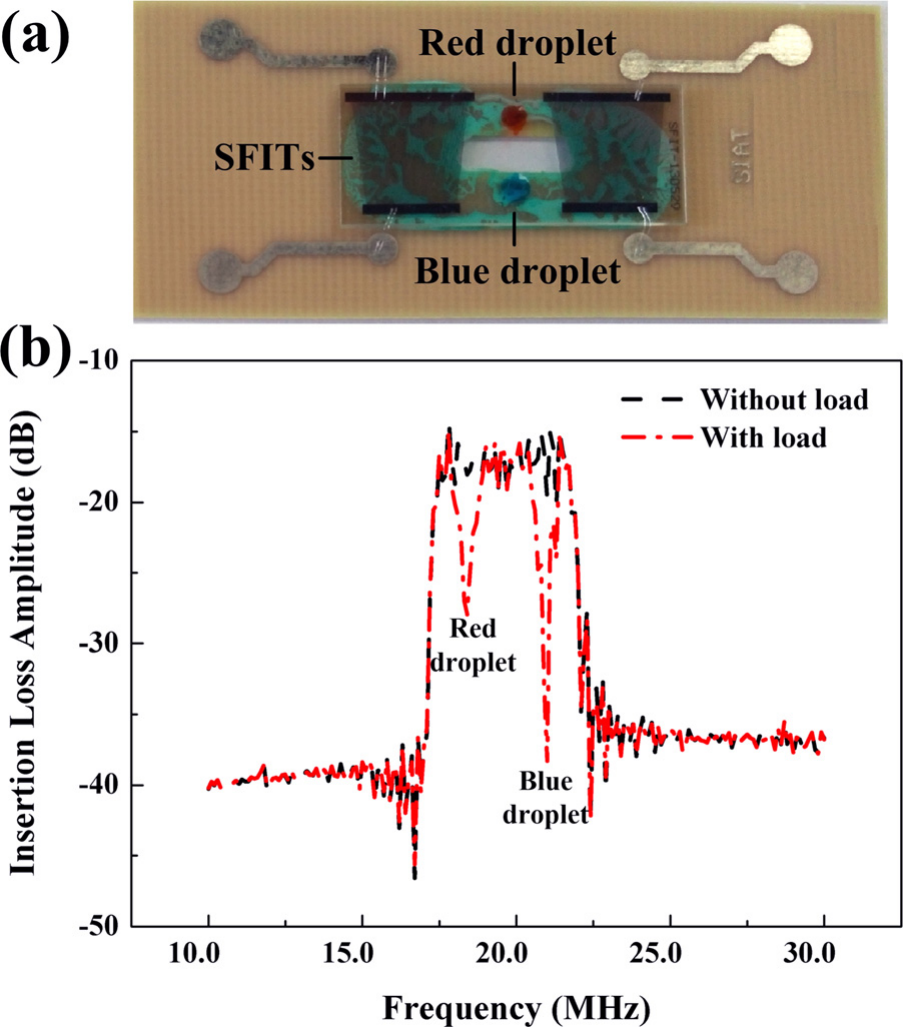
(a) Picture of the SFITs-based microfluidic device. (b) The insertion loss of this device from 17.10 MHz to 22.00 MHz. The black dashed line shows the insertion loss of the device without any load and the red dashed-dotted line represents the insertion loss of SFITs with two different sized droplets. The insertion loss of SFITs at 21.00 MHz is larger than that at 18.40 MHz as the blue droplet has the larger volume. The diameter of the red droplet and the blue droplet was 2.3 mm and 2.8 mm, respectively.

To validate the performance of the SFITs, a network analyzer (ZVA40 Rohde and Schwarz, Germany) was used to measure the insertion loss of the SFITs. Fig. 2(b) shows that the transmission spectrum of the SFITs is relatively broad and smooth (the black dashed line) from 17.10 MHz to 22.00 MHz, which is in good agreement with theoretical predictions. The insertion loss of the SFITs was about −17 dB without any load on the substrate. As shown in Fig. 2(a), two water droplets (red and blue) with different sizes were placed at different locations on the pathway of the SAWs propagation along the substrate. A greater sharp signal loss of the transmission spectrum (the red dashed-dotted line) was observed at 18.40 MHz and 21.00 MHz (Fig. 2(b)). These two frequencies matched with the resonance frequencies determined by two different portions of a pair of SFITs with different spacing between two nearest fingers along the Y axis, which was in agreement with the positions where the two droplets were located. Moreover, the insertion loss of the transmission spectrum at the 21.00 MHz was much higher than that of the 18.40 MHz because of the higher attenuation due to the larger volume of the blue droplet. The results indicate that there is a corresponding relationship between the position of the acoustic aperture along the Y direction and acoustic resonant frequency.

### B. Frequency-shift manipulation of MBs

**FIG. 3.**
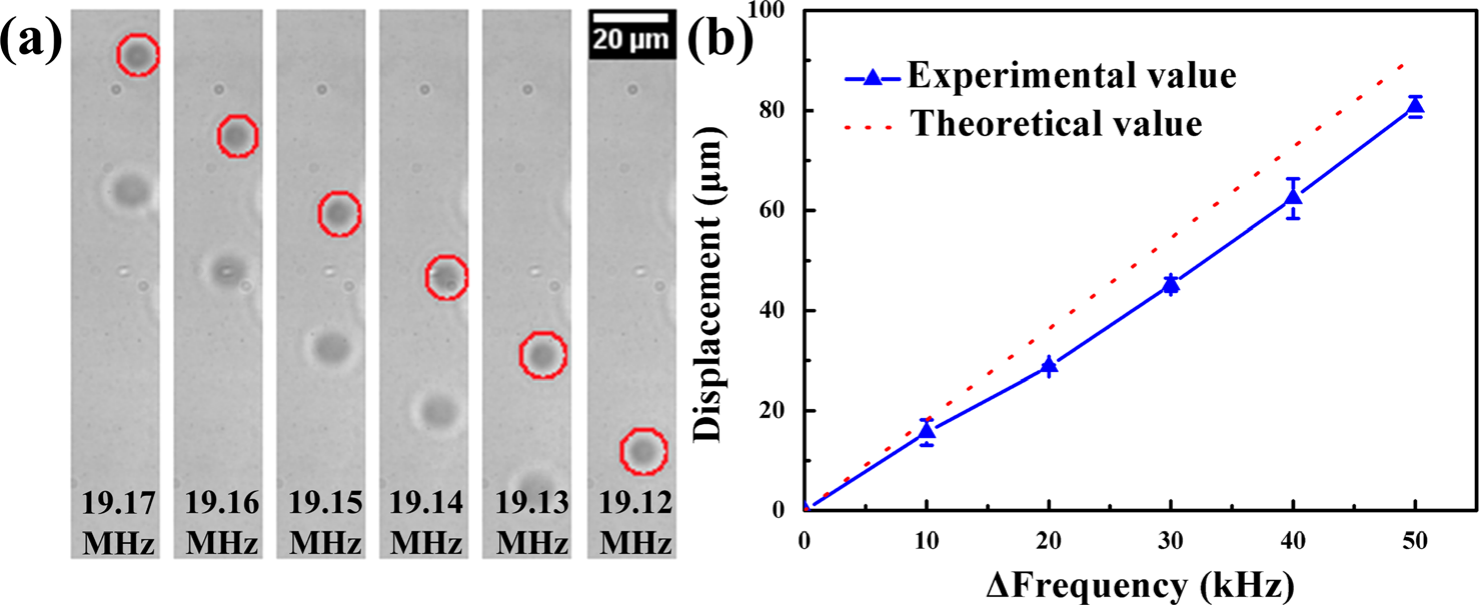
(a) The movement of MBs along the acoustic aperture by changing the input frequency from 19.17 MHz to 19.12 MHz at a step of 10 kHz. (b) The relationship between the displacement of the MBs and the shift of the frequency. (Multimedia view) [URL: http://dx.doi.org/10.1063/1.4954934.1]
10.1063/1.4954934.1

To investigate the frequency-shift effects in manipulating MBs in the direction perpendicular to acoustic propagation, the input signal frequency was changed continuously to excite the resonance of the SFITs at different locations. Initially, MBs were injected into a microchannel by a micro-syringe pump. The SFITs were connected to a power amplifier which amplifies a continuous sine-wave signal of 19.17 MHz. The MBs moved immediately and aggregated into clusters at the pressure nodes. When the input frequency was decreased at a step of 10 kHz, MBs moved toward the negative Y direction continuously. Fig. 3(a) (Multimedia view) shows the movement of the MBs in Y direction when changing the input signal frequency from 19.17 MHz to 19.12 MHz at a step of 10 kHz. The velocity of movement was nearly the same at each transportation, with the speed of 50 *μ*m/s.

The displacement of the MBs was processed by ImageJ software (National Institutes of Health, Bethesda, MD). Fig. 3(b) shows the result of movement of the MBs cluster as the function of the shift of input signal frequency. It is evident that the displacement of MBs agrees well with the theoretical analysis and there is a linear relationship between the displacement and the shift of driving frequency. This means that the MBs can be handled precisely in the Y direction and the resolution of displacement of the MBs can reach 15.19 ± 2.65 *μ*m with the frequency-shift of 10 kHz. Higher precision of resolution can also be achieved by decreasing the shift of the driving frequency (supplementary material, Figure S1). The change in the driving frequency of 10 kHz induced the translation of the pressure nodes which was too small to be detected in the X direction. During the above-mentioned experiment, it was noticed that the displacement of the MBs in X direction was negligible. Thus, by changing the driving frequency of SFITs, the MBs can be transported along the acoustic aperture (Y) direction perpendicular to acoustic propagation.

### C. Selective manipulation of polystyrene beads

**FIG. 4.**
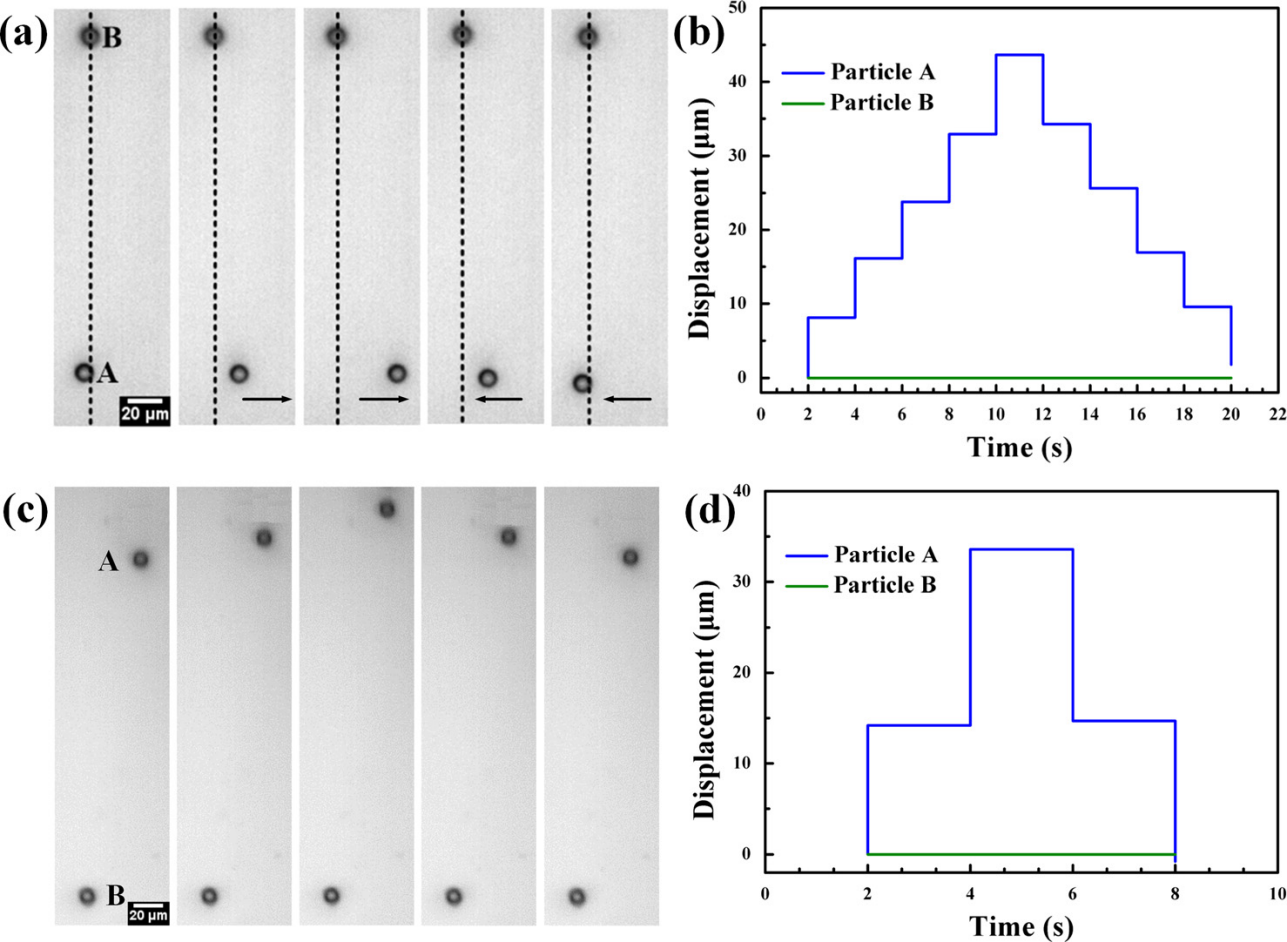
(a) Selective and specific manipulation of two identical particles. Particle A could be translated along the X direction by adjusting the relative phase between SFITs at 17.20 MHz. Particle B was located outside the resonant position and remained stationary. (b) The displacement of particles in the X direction as a function of time. (c) A particle can also be manipulated selectively in Y direction by adjusting the driving frequency. (d) The displacement of particles in the Y direction as a function of time. (Multimedia view) [URL: http://dx.doi.org/10.1063/1.4954934.2]
10.1063/1.4954934.2

To investigate the selective manipulation of a specific particle in the channel, two identical spherical beads with the same material and diameter have been injected into the microchannel. Two beads suspended in water and dispersed to different locations in the Y direction matched with different resonant frequencies. The resonant frequency corresponding to the position where the particle A located was approximately 17.20 MHz. Fig. 4(a) (Multimedia view) shows that the particle A was driven to move along the X direction when the continuous sinusoidal signal at 17.20 MHz was applied to the SFITs using the phase-shift method. By adjusting the relative phase between the two transducers at a step of 30° continuously, particle A could be translated precisely and eventually returned to the location where it started. Fig. 4(b) shows the track of the particle A by changing the relative phase at 17.20 MHz as the function of time. Particle B located outside the resonant location at 17.20 MHz remained stationary. However, by adjusting the relative phase between SFITs at 17.31 MHz, particle B could be moved immediately, indicating that particle B could not be driven by the SAWs at the frequency of 17.20 MHz rather than it adhered to the substrate. This observation demonstrates that the particles located anywhere in the microchannel can be selectively translated in the X direction by changing the relative phase at the corresponding resonant frequency.

Similarly, a single particle can be transported selectively in the Y direction by adjusting the driving frequency of the input RF signals. Fig. 4(c) shows that two identical particles suspend in the microchannel and the resonant frequencies corresponding to the positions of particle A and particle B located were about 18.89 MHz and 18.77 MHz, respectively. When the SFITs were applied with the frequency of 18.89 MHz, cluster A was activated and could be transported along the Y direction precisely. Particle B, on the other hand, remained stationary as it located outside of resonant location from 18.89 MHz to 18.91 MHz. The results indicate that the selective manipulation can be achieved in the both X and Y directions.

### D. Two-dimensional manipulation of MBs

**FIG. 5.**
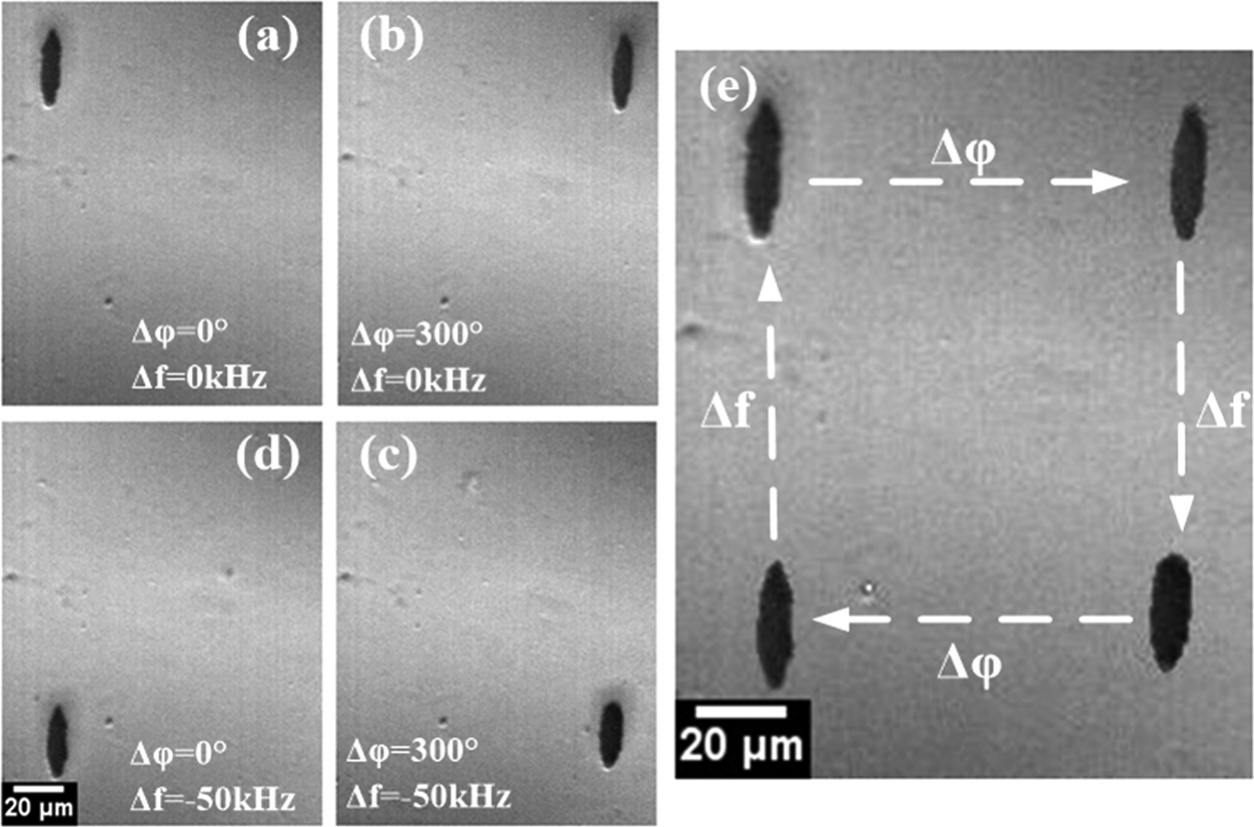
Flexible and dynamic manipulation of MBs. The MBs can be moved arbitrarily around a plane by a combination of phase-shift and frequency-shift methods together. (a)–(d) MBs were transported in a rectangle track by adjusting the relative phase from 0° to 300° and adjusting the input frequency from 17.17 MHz to 17.12 MHz. (e) The composite image shows the trajectory of MBs cluster.

Due to the linear relationship between the displacement and shift of frequency and relative phase, further experiments were carried out to demonstrate the flexibility and the robust nature of the manipulation in two dimensions by combining the change in driving frequency with relative phase together. As shown in Fig. 5, MBs cluster were moved along a rectangle trajectory. Setting the value of driving frequency to 17.17 MHz and Δφ to 0°, MBs were trapped at the pressure nodes and suspended at the top left corner (Fig. 5(a)). Increasing the Δφ to 300° at a step of 10° continuously, the MBs cluster moved toward the positive X-axis direction (Fig. 5(b)). When the driving frequency was decreased to 17.12 MHz continuously, MBs cluster moved toward the negative Y-axis direction (Fig. 5(c)). Adjusting the value of Δφ to 0°, MBs cluster moved along the negative X-axis direction (Fig. 5(d)). Finally, MBs could return to the starting position after resetting Δφ to 0° and driving frequency to 17.17 MHz (Fig. 5(a)). Fig. 5(e) is a composite image showing the four states of MBs cluster at different times. It is of note that the volume of the MBs cluster also had an influence on the precision of the transportation (supplementary material, Figure S2). The larger cluster had a relatively larger inertia weight and was more likely to deform in morphology under the action of acoustic radiation force, thus reducing the accuracy of manipulation. The results reveal that MBs can be transported anywhere in a plane with any arbitrary trajectory, facilitating the MBs continuous movement in a large scale. Therefore, flexible and dynamic manipulation of MBs can be achieved on this one-dimensional device using this combination method.

## IV. CONCLUSIONS

In summary, we have demonstrated a flexible technology which can manipulate MBs' positions selectively in two dimensions by using a pair of SFITs. The resonant frequency of the SFITs changes linearly along the acoustic aperture (Y) direction. Only the particles located at the resonant position can be translated in the X direction by the phase-shift method. Furthermore, the particle also can be transported selectively along the Y direction by changing the corresponding driving frequency of SFITs continuously. The good linear relationship between the displacement and shift of frequency enables transport of the MBs precisely. The resolution of transportation based on frequency-shift method is approximately 15.19 ± 2.65 *μ*m in Y direction when the shift of the input signal frequency is 10 kHz. By combining frequency-shift with phase-shift, this SFITs device may be used in MBs transportation in two dimensions. This device opens the door for applications in selective and dynamic manipulation of particles relevant to tissue engineering and targeted drug delivery.

## SUPPLEMENTARY MATERIAL

See supplementary material for the displacement resolution of microbubbles (MBs) at the shift of frequency of 5 kHz (Figure S1) and the relationship between the volume of the microbubble cluster and the displacement in X and Y axes (Figure S2).
